# Clinical impact of digital and conventional PET control databases for semi-quantitative analysis of brain ^18^F-FDG digital PET scans

**DOI:** 10.1186/s13550-020-00733-y

**Published:** 2020-11-30

**Authors:** Elise Mairal, Matthieu Doyen, Thérèse Rivasseau-Jonveaux, Catherine Malaplate, Eric Guedj, Antoine Verger

**Affiliations:** 1 Department of Nuclear Medicine and Nancyclotep Imaging Platform, CHRU Nancy, Université de Lorraine, Rue du Morvan, 54500 Vandoeuvre-les-Nancy, France; 2 IADI, INSERM U1254, Université de Lorraine, 54000 Nancy, France; 3 Clinical Memory and Research Center, Department of Geriatrics, CHRU Nancy, Université de Lorraine, 2LPN EA 7489, 54000 Nancy, France; 4 Department of Biochemistry, Molecular Biology and Nutrition CHRU Nancy, Université de Lorraine, 54000 Nancy, France; 5 APHM, CNRS, Centrale Marseille, Institut Fresnel, Timone Hospital, CERIMED, Nuclear Medicine Department, Aix Marseille Univ, Marseille, France

## Abstract

**Purpose:**

Digital PET cameras markedly improve sensitivity and spatial resolution of brain ^18^F-FDG PET images compared to conventional cameras. Our study aimed to assess whether specific control databases are required to improve the diagnostic performance of these recent advances.

**Methods:**

We retrospectively selected two groups of subjects, twenty-seven Alzheimer's Disease (AD) patients and twenty-two healthy control (HC) subjects. All subjects underwent a brain ^18^F-FDG PET on a digital camera (Vereos, Philips®). These two group (AD and HC) are compared, using a Semi-Quantitative Analysis (SQA), to two age and sex matched controls acquired with a digital PET/CT (Vereos, Philips®) or a conventional PET/CT (Biograph 6, Siemens®) camera, at group and individual levels. Moreover, individual visual interpretation of SPM T-maps was provided for the positive diagnosis of AD by 3 experienced raters.

**Results:**

At group level, SQA using digital controls detected more marked hypometabolic areas in AD (+ 116 cm^3^ at *p* < 0.001 uncorrected for the voxel, corrected for the cluster) than SQA using conventional controls. At the individual level, the accuracy of SQA for discriminating AD using digital controls was higher than SQA using conventional controls (86% vs. 80%, *p* < 0.01, at *p* < 0.005 uncorrected for the voxel, corrected for the cluster), with higher sensitivity (89% vs. 78%) and similar specificity (82% vs. 82%). These results were confirmed by visual analysis (accuracies of 84% and 82% for digital and conventional controls respectively, *p* = 0.01).

**Conclusion:**

There is an urgent need to establish specific digital PET control databases for SQA of brain ^18^F-FDG PET images as such databases improve the accuracy of AD diagnosis.

## Introduction

Digital PET cameras are composed of small digital silicon photomultipliers, which provide digital photon counting with a 1-to-1 crystal coupling, and replace the larger photomultiplier tubes of conventional PET cameras [1, 2]. These digital PET cameras thus provide improvements in detection sensitivity, spatial resolution and signal to noise ratio and therefore in image quality compared to conventional PET cameras. This has been a particularly significant advance in brain ^18^F-FDG PET acquisitions [1, 2].

Brain ^18^F-FDG PET is a useful tool for diagnosing neurodegenerative disorders. It is particularly useful in the diagnosis of Alzheimer’s Disease (AD) [3], where a visual analysis of brain ^18^F-FDG PET images is initially performed to detect the typical AD hypometabolic pattern involving the posterior temporo-parietal association cortex [4, 5]. Semi-quantitative analysis (SQA) has been proposed as an adjunct to this visual analysis since it increases confidence in the diagnostic conclusion drawn, particularly at earlier stages of the disease [6]. The importance of SQA has been further underpinned by its inclusion in the recommendations of the European Association of Nuclear Medicine and European Academy of Neurology [3].

The establishment of a well-documented reference database is a crucial step for performing SQA. A comparison to homogeneous control population databases is an important cornerstone to reduce false positives. Indeed, age and sex effects have been reported to influence metabolism distribution in brain ^18^F-FDG PET images [7]. In addition, all currently implemented control databases in dedicated software for automated SQA are still based on acquisitions performed with conventional PET [8–13]. It is very likely that improvements in image quality provided by digital PET technology may also influence results of SQA, even though, to the best of our knowledge there is currently no data in the literature which evaluates the potential clinical impact of such an effect.

Our current study aims to assess whether there is a need to establish specific reference control databases which take into account the recent technical advances of SQA on brain ^18^F-FDG PET images with respect to the evolution of digital PET.

## Materials and methods

### Subjects

We retrospectively selected two groups of subjects, a group of patients with AD and a group of healthy control subjects (HC). Both groups had undergone a brain ^18^F-FDG PET scan on a digital PET/CT (Vereos, Philips®), at the CHRU of Nancy, France, between December 2017 and September 2019.

The AD patients selected fulfilled the NIA-AA 2018 criteria for AD [14]. They exhibited positive cerebro-spinal fluid biomarkers with increased phosphorylated Tau protein and reduced beta-amyloid peptide levels measured in the same laboratory (Department of Biochemistry, Molecular Biology and Nutrition, CHRU Nancy, France) using standard cut-offs [15]. AD patients also underwent a routine neurocognitive assessment in the “memory clinic” of the university hospital of Nancy (France).

Healthy control subjects were age and sex matched with patients in the AD group and were also selected retrospectively. All healthy control subjects had undergone a brain ^18^F-FDG PET scan for cognitive assessment, but had returned a normal scan by careful visual analysis (EM, AV) and a neuropsychological assessment which was not consistent with a neurodegenerative disorder: (i) normal neuropsychological tests, i.e. MMSE ≥ 27, FAB ≥ 15 and no major depressive disorders and (ii) a clinical follow-up, of longer than 1 year, which showed a stabilisation and/or improvement of cognitive symptoms.

**Fig. 1:**
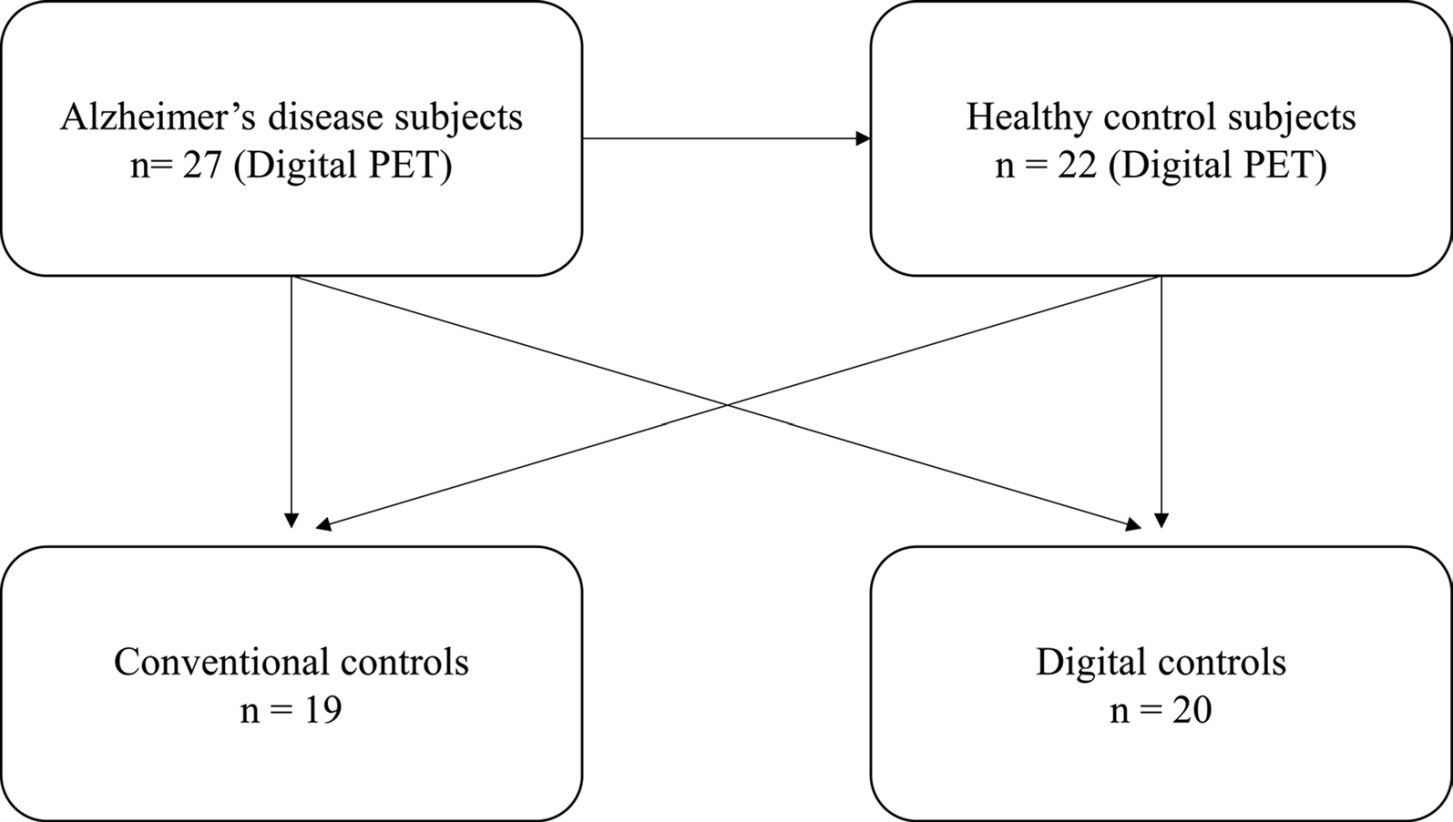
Flowchart for the constitution of the two different groups of Alzheimer’s disease and healthy control subjects, and the conventional and digital control databases. Black arrows are representative of analyses performed

Our AD and HC groups were further compared to each other and to two control databases (a digital and a conventional database) derived from prospective studies. Individuals from these two control databases had undergone a brain ^18^F-FDG PET/CT performed with a conventional camera between October 2009 to May 2012 (*n* = 19, Biograph 6, Siemens®, NCT02858167) or performed with a digital camera between December 2017 to June 2019 (*n* = 20, Vereos, Philips®, NCT03345290) and were age and sex matched with our AD and HC groups. A flowchart summarising the constitution of the different control groups is shown in Fig. 1.

Informed consent was obtained for each participant included in the selected groups. This study was approved on January 16, 2020 by the local ethics committee (NCT04163276, Study ID Numbers: 2019PI238).

### *Brain *^*18*^*F-FDG PET*

The brain ^18^F-FDG PET scan was recorded over a 10 (for conventional camera) to 15 min (for digital camera) one bed acquisition, 45–50 min after injection of 4.5 MBq/kg (conventional camera) or 2–3 MBq/kg (digital camera) of ^18^F-FDG. All subjects had fasted at least 6 h prior to receiving the injection and had blood glucose levels < 10 mmol/L. All PET images were reconstructed with iterative OSEM methods, as performed in routine clinical practice, and corrected for scatter, random and attenuation with a CT scan. Reconstructed parameters included 4 iterations and 8 subsets, subsequently displayed in a 168 × 168 matrix with 2.7 × 2.7 × 2.7 voxels for the conventional PET camera [7], and 3 iterations and 15 subsets, subsequently displayed in a 256 × 256 matrix with 1 × 1 × 1 mm^3^ voxels for the digital PET camera [2].

### Statistical parametric mapping

The ^18^F-FDG PET brain images were pre-processed using SPM12 (Wellcome Department of Cognitive Neurology, Institute of Neurology, London, UK) running on Matlab 2018a (MathWorks Inc., Sherborn, MA). After an initial step of approximate manual re-orientation and positioning to MNI space, the spatial normalisation of each PET image into the MNI space was performed by spatial normalisation of the CT scan for each subject provided by the correction of attenuation, using the method and the template of the Clinical Toolbox for SPM (https://www.nitrc.org/projects/clinicaltbx/). The voxel sizes of the written CT images were set to 1 × 1 × 1 mm^3^ for the digital controls and to 2 × 2 × 2 mm^3^ for the conventional controls. Each CT spatial normalisation procedure was subsequently applied to the respective PET images. Voxels of PET images recorded with the conventional camera during this step were therefore resampled from 2.7 × 2.7 × 2.7 mm^3^ to 2 × 2 × 2 mm^3^ whereas voxel sizes of the digital databases were not modified (1 × 1 × 1 mm^3^). To enable voxel-to-voxel analysis with these two control databases, AD and HC group datasets were normalised using the two voxel sizes. Partial volume effect corrections were applied to PET images using the Müller-Gärtner (MG) method provided by the PETPVE12 toolbox [16]. White and grey matter segmentations needed for the 3-compartmental voxel-wise MG method were realised on CT scans using SPM segmentation tools, after careful visual analysis at the individual level to check the accuracy of this segmentation. The cerebellum was used as reference for the intensity normalisation of PET images because normalisations other than the proportional scaling have been proposed [17] and because the cerebellum is associated with a more accurate discrimination of patients with AD compared to controls [18]. All regions of interest (ROIs) used for intensity normalisation (all the cerebellum and vermis ROIs for the cerebellum, and all the pre- and post-central brain areas for the sensorimotor cortex) were extracted from the AAL atlas [19] after spatial normalisation to limit the inter-individual anatomical heterogeneity. Finally, PET images were smoothed with an isotropic 3D Gaussian kernel of 12 mm FWHM to blur individual variations in gyral anatomy. Visual inspections of the images at the different stages of the pre-processing procedure ensured the quality and convergence of the different methods applied.

Semi-Quantitative Analyses (SQA) were performed at the group and individual level on a voxel-by-voxel basis using two-sample t-tests with an inclusive AD mask [20]. At the group level, AD and HC groups were compared with the conventional and digital controls using age and sex as covariates (clusters of decreased metabolic activity observed at *p* < 0.001 for the voxel, cluster volume corrected by using the expected volume provided by SPM and based on the random field theory). We used exclusive masks to compare results obtained with SQA to conventional or digital controls. For AD, an exclusive mask corresponding to the SPM-T map results of SQA to conventional controls was applied to the SQA of digital controls to highlight the additional clusters visualised with the digital system compared to the conventional system (and vice versa for the HC population).

At the individual level, each subject in the AD and HC group, was individually compared to the digital and conventional controls using a fully automated analysis as well as visual analyses (clusters of decreased metabolic activity observed at *p* < 0.005 for the voxel, cluster volumes corrected to 0.8 cm^3^ [6, 21]). All clusters identified with SPM at the individual level were considered significant for the fully automated analysis.

The precise identification of each structure located by its MNI coordinates, its respective volume, and T-max intensity were extracted by using the report provided by the SPM xjView toolbox (http://www.alivelearn.net/xjview).

### Visual ratings at the individual level

For the visual analyses, the SPM T-maps were projected onto three-dimensional rendering of T1-weighted MRI images using SPM surface rendering tool and onto 12 two-dimensional slices of T1-weighted MRI images using the Slice Display toolbox [22] (axial orientation, inter-slice spacing of 1 cm). Representations were reviewed by three experienced observers (EM, EG and AV), who were blinded to the patient’s clinical data. Raters were forced to give a dichotomous reading: Alzheimer’s disease diagnosis or not pathological. A pattern of diffuse hypometabolic areas within the areas known to be involved in AD (mainly the bilateral posterior associative areas) was considered a positive scan. At the individual level, results were expressed as a consensual analysis for the positive diagnosis of AD.

### Statistical analysis

Categorical variables are expressed as percentages and continuous variables as means and standard deviations. Due to the non-normality of variable distributions, Chi-2 and Kruskal–Wallis tests were performed for comparisons of categorical and continuous variables, respectively. For the comparisons of diagnostic performances at the individual level, Mc Nemar tests were used with corrections for multiple comparisons. A *p* value < 0.05 was considered as significant. All tests were performed with SPSS (SPSS Statistics for Windows, Version 20.0. Armonk, NY: IBM Corp).

## Results

### Population

As detailed in Table 1, no difference in age, sex and educational level were observed between the AD, HC groups, as well as the conventional and digital controls (*p* > 0.27). As expected, AD patients showed lower levels of MMSE than HC subjects as well as conventional and digital controls (*p* < 0.01).

**Table 1:** Characteristics of the Alzheimer's disease patients, healthy control subject’s as well as the conventional and digital control groups

Group	AD (*n* = 27)	HC (*n* = 22)	Conventional controls (*n* = 19)	Digital controls (*n* = 20)	*p* values
Age (years old)	70.9 ± 7.5	67.7 ± 10.4	67.2 ± 8.3	66.6 ± 8.5	0.27
Sex (Female)	14 (52%)	10 (45%)	8 (42%)	8 (40%)	0.86
Educational level					0.60
None	1	0	0	0	
Primary school	11	9	14	12	
High school	4	3	2	3	
College	11	9	3	5	
NA	–	1	–	–	
MMSE	19.2 ± 5.7*	28.7 ± 0.9	29.2 ± 0.8	29 ± 0.9	< 0.01*

AD, Alzheimer’s disease; HC, healthy control; MMSE, mini-mental state examination score

*p < 0.05 for comparisons between groups

### At the group level

In patients with AD, SQA using digital controls enabled the detection of more marked hypometabolic areas (+ 116 cm^3^) when compared to SQA using conventional controls. There were 3 more clusters of significance identified with SQA using digital controls. The most extensive one involved the bilateral posterior associative areas, including the precuneus and the posterior cingulate (T-voxel max at 7.88). The two others, less extensive and with lower T-voxel max values involved the bilateral anterior associative areas.

**Fig. 2:**
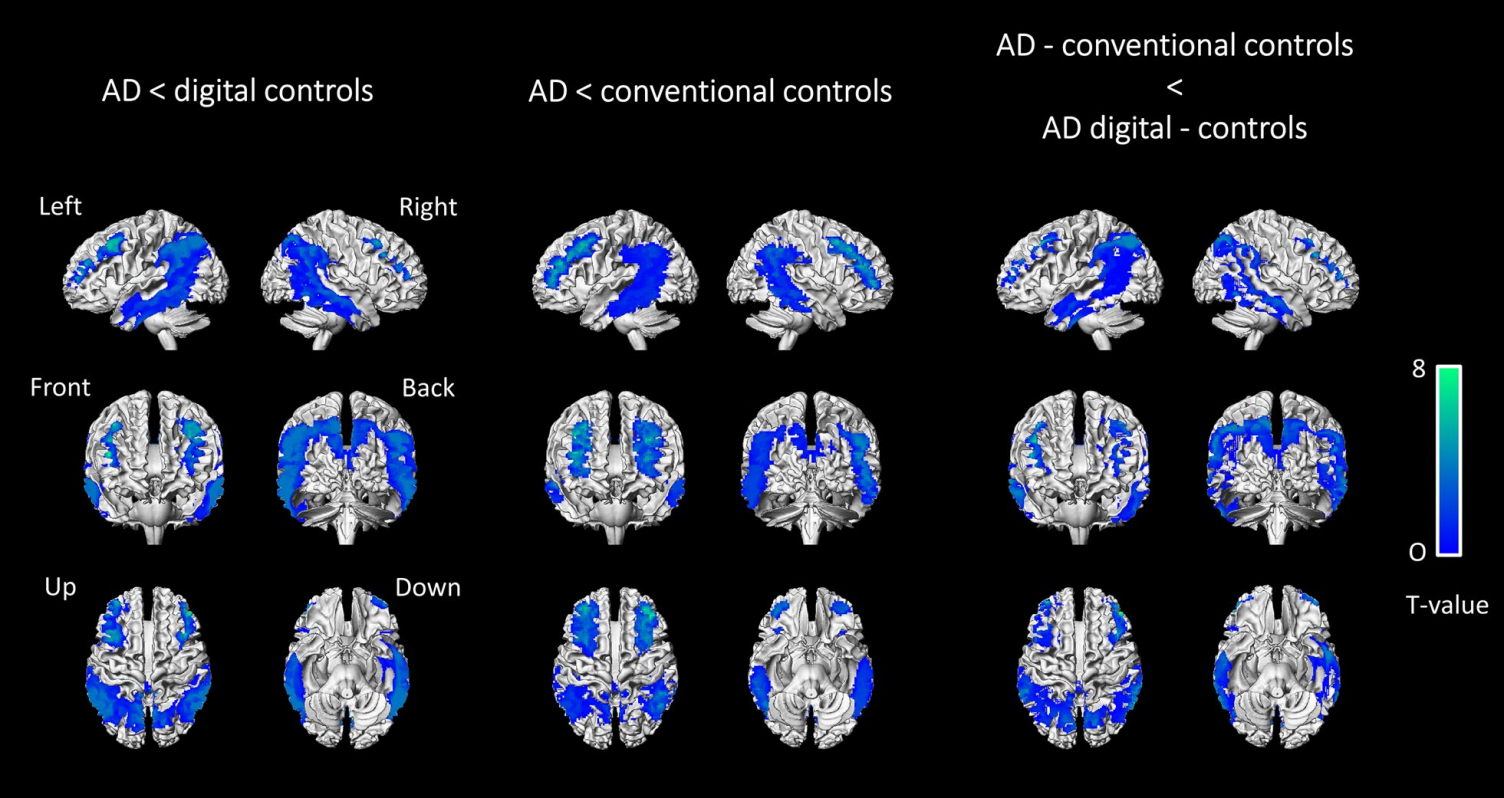
Anatomical localisation of areas of decreased metabolic activity in AD patients (*p* < 0.001, uncorrected, *k* > 1.86 cm^3^) for SQA using digital controls (left panel), SQA using conventional controls (middle panel), and the significant additional hypometabolic areas obtained with SQA using digital controls compared to SQA using conventional controls (right panel), projected onto 3D volume rendering, spatially normalised and smoothed into the standard SPM template (T-voxel threshold value of 3.3)

Details of significant additional hypometabolic regions obtained with SQA using digital controls in patients with AD are provided in Table 2 and Fig. 2.

**Table 2:** Additional AD patient clusters identified with SQA using digital controls as opposed to SQA using conventional controls (anatomical locations, spatial extent of significant clusters in cm^3^, MNI coordinates, maximal T-scores of the peak voxel) at a T-voxel threshold of 3.3, k cluster size > 1.86 cm^3^

Anatomical location	Cluster size	x	y	z	T-score of peak
Right and left cerebrum					
Cuneus Precuneus Posterior cingulate Superior parietal Inferior parietal Superior temporal Middle temporal Inferior temporal Superior occipital Middle occipital Inferior occipital	105	− 33	− 10	− 44	7.88
Right cerebrum					
Superior frontal Middle frontal Inferior frontal	4	30	57	− 2	5.29
Left cerebrum					
Superior frontal Middle frontal Inferior frontal	7	− 36	55	0	4.16

**Fig. 3:**
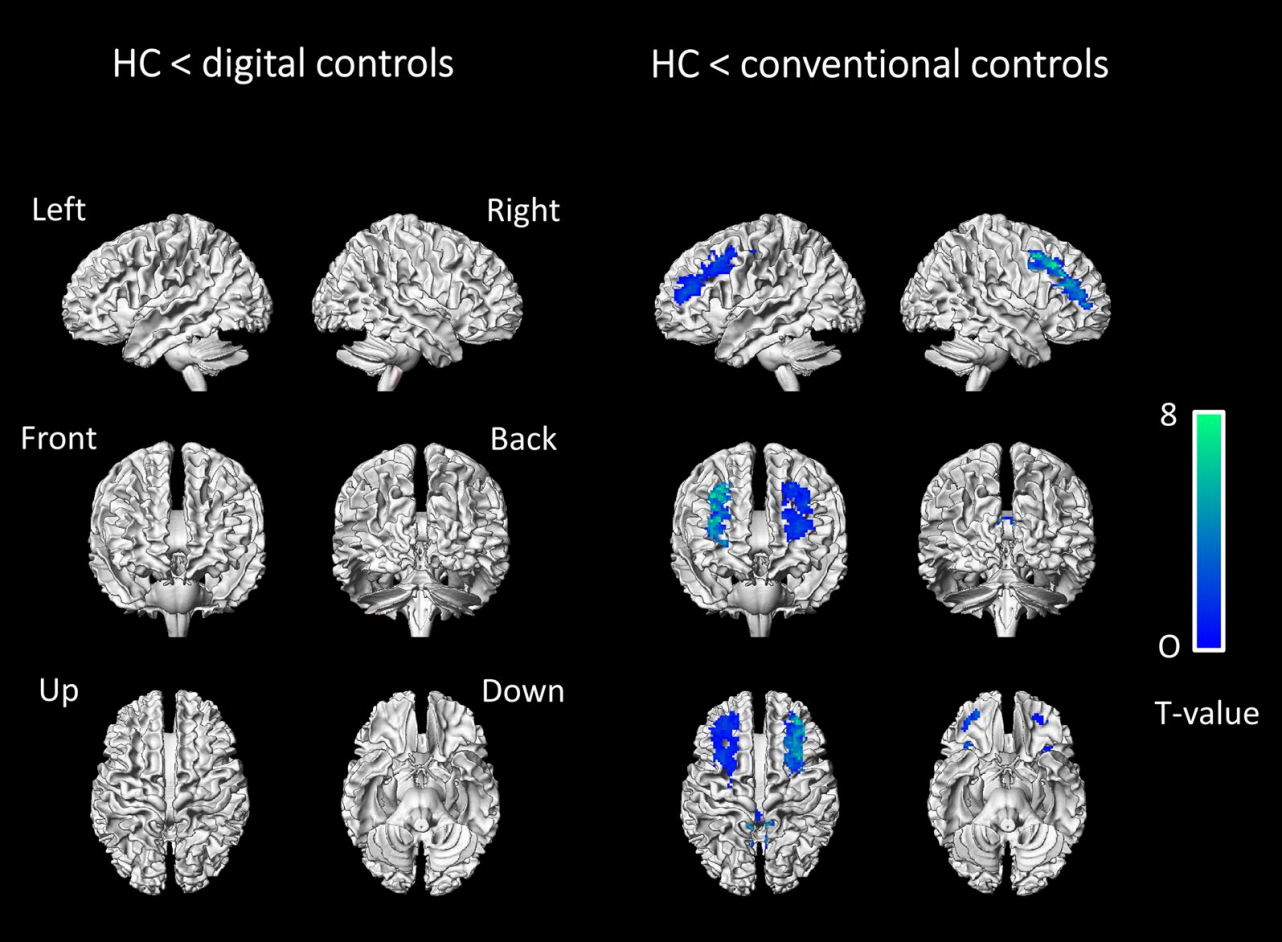
Anatomical localisation of areas of decreased metabolic activity in HC subjects (*p* < 0.001, uncorrected, *k* > 1.45 cm^3^), with SQA using digital controls (left panel) and SQA using conventional controls (right panel), projected onto 3D volume rendering, spatially normalised and smoothed into the standard SPM template (T-voxel threshold value of 3.3)

Moreover, in healthy control subjects (HC), SQA using conventional controls showed more marked hypometabolic areas (+ 17 cm^3^) when compared to SQA using digital controls. There were 3 more significant clusters identified with SQA using conventional controls involving the precuneus, and the bilateral anterior associative areas. Details of significant hypometabolic areas obtained with SQA using conventional controls in healthy control subjects are provided in Table 3 and Fig. 3.

**Table 3:** Additional HC patient clusters identified with SQA using conventional controls as opposed to SQA using digital controls (anatomical locations, spatial extent of significant clusters in cm^3^, MNI coordinates, maximal T-scores of the peak voxel) at a T-voxel threshold of 3.3, k cluster size > 1.45 cm^3^

Anatomical location	Cluster size	x	y	z	T-score of peak
Right cerebrum					
Middle frontal	5	34	2	38	5.73
Left cerebrum					
Middle frontal	7	− 24	2	46	5.01
Right cerebrum					
Precuneus	5	10	− 52	22	5.96

Of note, significant hypermetabolism was also shown with SQA using conventional controls both for comparisons to patients with AD (+ 21 cm^3^) and to HC subjects (+ 115 cm^3^) whereas no significant hypermetabolism for these comparisons was observed for SQA using digital controls.

For further validation, the AD and HC groups, both acquired with the same digital PET scanner, were compared at the group-level. The volume of cluster significance was similar to that observed in SQA using digital controls for patients with AD (195 cm^3^ with a T-voxel max value of significance at 7.48, vs. 185 cm^3^ with a T-voxel max value of significance at 7.88 for the comparison between patients with AD and the digital controls).

### At the individual level

The accuracy of SQA for discriminating AD using digital controls was higher than with conventional controls (86 vs. 80%), with a higher sensitivity (89 vs. 78%) and similar specificity (82% vs. 82%) achieved with the fully automated analysis.

These results were confirmed by the visual analysis with an accuracy, sensitivity and specificity of respectively 84%, 85%, 82% for SQA using digital and 82%, 67%, 100% for SQA using conventional controls.

Detailed diagnostic performances for both SQA using conventional and digital controls are reported in Table 4.

**Table 4:** Diagnostic performances of SQA using conventional and digital controls at the individual level with fully automated and visual analyses

	Accuracy (%)	Sensitivity (%)	Specificity (%)	*p* value
Fully automated				< 0.01*
SQA using conventional controls	80	78	82	
SQA using digital controls	86	89	82	
Visual				= 0.01*
SQA using conventional controls	82	67	100	
SQA using digital controls	84	85	82	

^*^For the comparison between SQA using conventional and digital controls

**Fig. 4:**
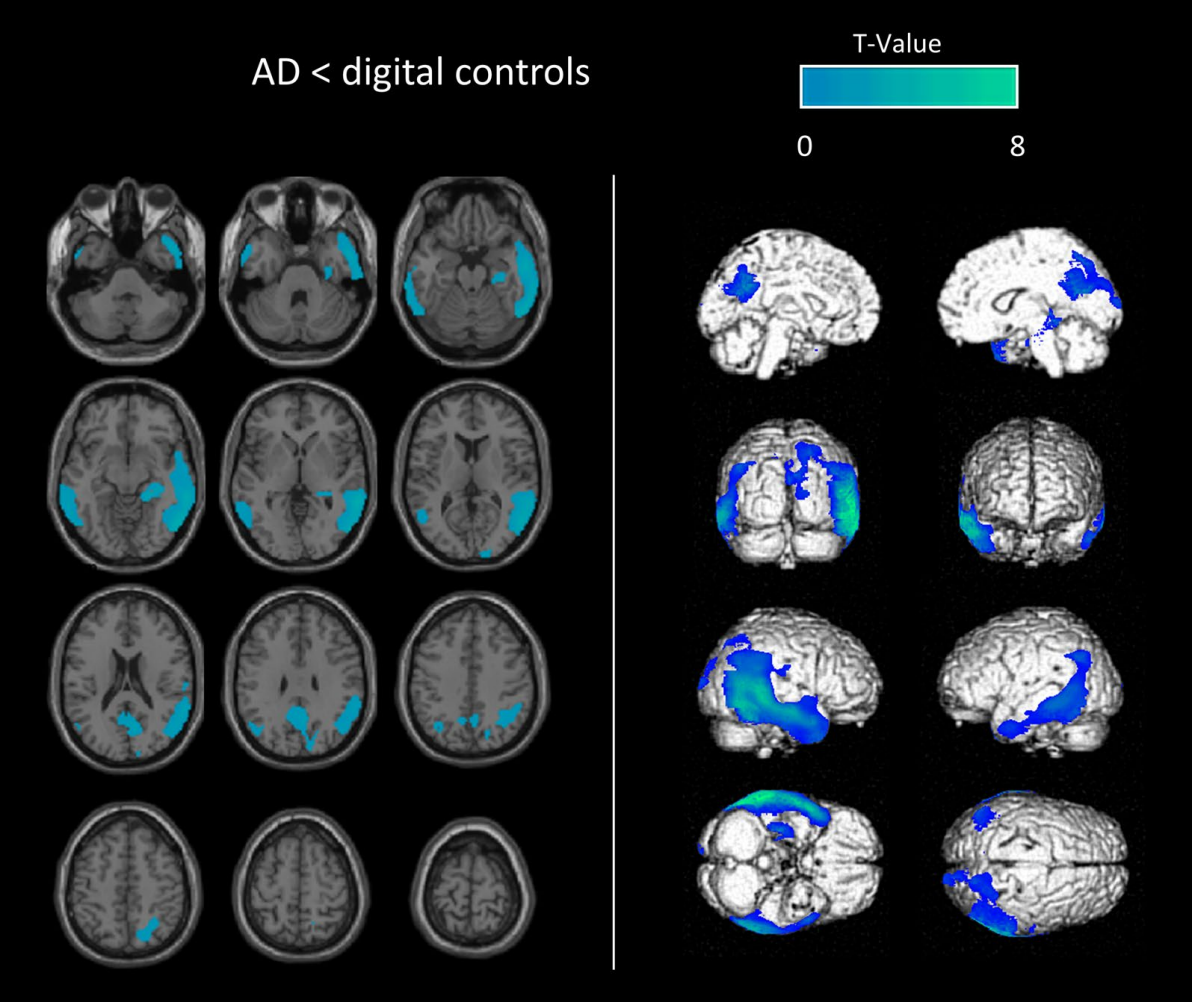
Example of SPM-T map images used for the visual analysis (semi-quantitative analysis to digital controls) of a patient with AD (79-year-old man with MMSE score of 10). SPM-T maps are projected onto two-dimensional slices of T1-weighted MRI (from the base to the top of the skull, left panel) and 3D-rendered volumes (right panel)

**Fig. 5:**
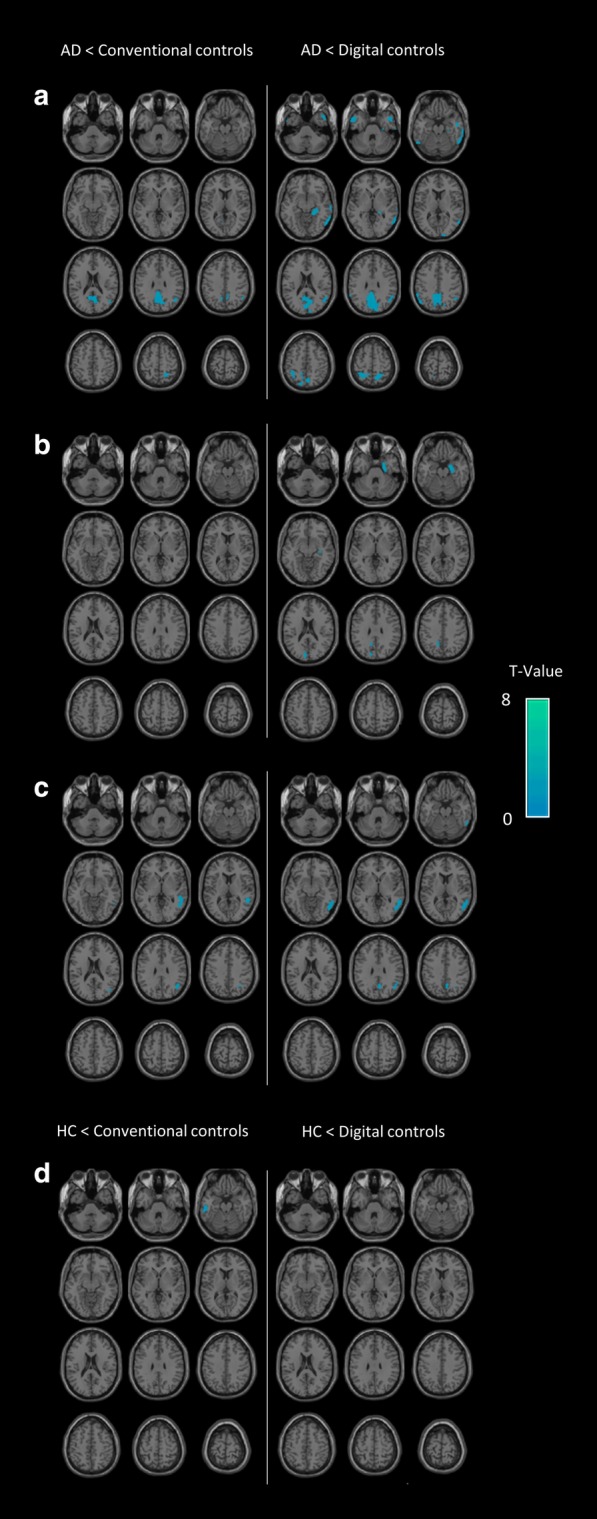
Galleria of individual examples with both semi-quantitative analyses to conventional and digital controls. Three examples of SPM-T map images from semi-quantitative analyses to conventional (left panel) and digital (right panel) controls of patients with AD (**a** 74-year-old woman with MMSE score of 19, **b** 70-year-old woman with MMSE score of 21, **c** 66-year-old man with MMSE score of 23). One example of SPM-T map images from semi-quantitative analyses to conventional (left panel) and digital (right panel) controls from a healthy subject (**d** 54-year-old woman with MMSE score of 29). SPM-T maps are projected onto two-dimensional slices of T1-weighted MRIs (from the base to the top of the skull)

An illustration of SPM-T map images used for the visual analysis is displayed in Fig. 4. Figure 5 provides a galleria of individual patients with both SQA to conventional and digital controls.

## Discussion

This study aimed to assess whether the event of SQA and the evolution of digital PET imaging requires the control databases to evolve concomitantly. Diagnostic performances observed at the group and individual level show that the diagnostic accuracy of SQA on digital controls is improved compared to SQA on conventional controls, particularly as it relates to the detection sensitivity of AD. This observation is an argument which supports the development of digital control databases for SQA of brain ^18^F-FDG PET images for clinical practice.

Digital PET technology is associated with improvements in image quality, specifically better spatial resolution and signal-to-noise ratios compared to conventional PET cameras [2]. These distinct image qualities lead to problematic head-to-head comparisons between digital and conventional PET images as reflected in our study by the relatively poor diagnostic performance obtained for SQA using conventional controls for discriminating AD. However, all currently implemented control databases in dedicated software for automated SQA in clinical practice still rely on conventional PET control images [9, 13]. Of course, it is now possible to implement local databases in the majority of these types of software, but establishing control databases acquired with digital PET technology remains an extensive undertaking, particularly because it involves a relative recently implemented technology.

Our present study shows that implementing a control database acquired with digital PET technology yields an increase in the detection sensitivity of AD patients, not only at the group level (+ 116 cm^3^ of detected hypometabolism volume, Table 2) but also at the individual level (sensitivity of detection increased from 78 to 89%, Table 4). A high detection sensitivity is primordial in the diagnosis of AD since ^18^F-FDG PET is a biomarker of neurodegeneration, which contributes to the ATN classification, the N biomarker being directly associated with cognitive impairment in patients suspected to have neurodegenerative diseases [14].

All results in the current study were initially obtained using a fully automated analysis, which supports the objective nature of our observations in both the group and individual level analyses. This original fully automated methodology, which necessitated an adaptation of the levels of significance to detect anomalies, was exclusively based on the SPM software. From a clinical standpoint and at the individual level, this fully automated analysis was nevertheless consistent with the visual analysis, using a methodology that is very similar to that applied in previously published SQA studies [6, 12]. By using this visual analysis, the diagnostic performance of SQA for discriminating AD with the digital controls observed in our study (accuracy, sensitivity and specificity of respectively 84, 85 and 82%) was within the range of previously reported results (70–97.5% for accuracy, 62.3–96% for sensitivity and 84–99% for specificity) [6, 8–12, 23–25].

SQA at the individual level was also performed using an intensity normalisation based on the sensorimotor cortex, which has been suggested to improve diagnostic performance of SQA [26]. The finds results were comparable to intensity normalisation on cerebellum. SQA on digital controls show respectively accuracy, sensitivity and specificity 84%, 89% and 77% (vs. 86%, 89% and 82%). SQA on conventional controls show respectively accuracy, sensitivity and specificity 73%, 52% and 100% (vs. 80% 78% and 82%).

The post-filter used (Gaussian kernel of 12 mm FWHM) can be decreased relative to the size of the voxels used particularly for digital PET scans. However, when smoothing images with a Gaussian kernel of 4 mm FWHM, the accuracy to detect AD, respectively versus digital and conventional controls, remained unchanged (86% vs. 86% and 82% vs. 80%).

The main limitation of our study results from the fact that controls included in the conventional and digital control databases were different individuals. From an ethical perspective, it remains problematic to establish control databases acquired in parallel with both the conventional and digital PET systems, even if it would be feasible to scan the same set of HC subjects within a few months on two different scanners using the half-dose permitted by the high sensitivity of the digital PET. It should however be noted that controls included in our conventional and digital databases did not exhibit any differences in age, sex, MMSE and educational level when compared to each other, or when compared to the AD and HC groups. In addition, these two, distinct conventional and digital control databases are representative samples from current daily clinical practice. The main objective of the current study was to assess whether there is indeed a requirement to establish digital control databases when acquisitions are performed with the new digital PET system. A secondary issue that may be addressed is that the sample size of conventional and digital control databases is rather small (*n* = 19 and 20). This number of controls is nevertheless known to be sufficient to accurately perform group analyses with SPM [27].

Overall, in light of recent digital PET technology developments and considering that SQA is now clearly recommended for brain ^18^F-FDG PET image analysis, there is an urgent need to establish digital PET control databases for SQA of brain ^18^F-FDG PET images. This would be particularly helpful for improving the sensitivity required to detect AD patients. Large healthy control databases should be constituted and shared through the multicentre community using standardised imaging protocols.

## Data Availability

The data that support the findings of this study are available on request from the corresponding author (AV).
